# Protein/Peptide Aggregation and Amyloidosis on Biointerfaces

**DOI:** 10.3390/ma9090740

**Published:** 2016-08-30

**Authors:** Qi Lu, Qiuhan Tang, Yuting Xiong, Guangyan Qing, Taolei Sun

**Affiliations:** 1 State Key Laboratory of Advanced Technology for Materials Synthesis and Processing, Wuhan University of Technology, 122 Luoshi Road, Wuhan 430070, China; zjtxlq2005@whut.edu.cn (Q.L.); xiongyuting6567@whut.edu.cn (Y.X.); 2 School of Chemistry, Chemical Engineering and Life Science, Wuhan University of Technology, 122 Luoshi Road, Wuhan 430070, China; tangqiuhan@whut.edu.cn

**Keywords:** protein aggregation, neurodegenerative disease, biomaterials, biointerface, surface chemistry

## Abstract

Recently, studies of protein/peptide aggregation, particularly the amyloidosis, have attracted considerable attention in discussions of the pathological mechanisms of most neurodegenerative diseases. The protein/peptide aggregation processes often occur at the membrane–cytochylema interface in vivo and behave differently from those occurring in bulk solution, which raises great interest to investigate how the interfacial properties of artificial biomaterials impact on protein aggregation. From the perspective of bionics, current progress in this field has been obtained mainly from four aspects: (1) hydrophobic–hydrophilic interfaces; (2) charged surface; (3) chiral surface; and (4) biomolecule-related interfaces. The specific physical and chemical environment provided by these interfaces is reported to strongly affect the adsorption of proteins, transition of protein conformation, and diffusion of proteins on the biointerface, all of which are ultimately related to protein assembly. Meanwhile, these compelling results of in vitro experiments can greatly promote the development of early diagnostics and therapeutics for the relevant neurodegenerative diseases. This paper presents a brief review of these appealing studies, and particular interests are placed on weak interactions (i.e., hydrogen bonding and stereoselective interactions) that are also non-negligible in driving amyloid aggregation at the interfaces. Moreover, this paper also proposes the future perspectives, including the great opportunities and challenges in this field as well.

## 1. Introduction

### 1.1. Role of Protein/Peptide Aggregation in Daily Life

**Scheme 1:**
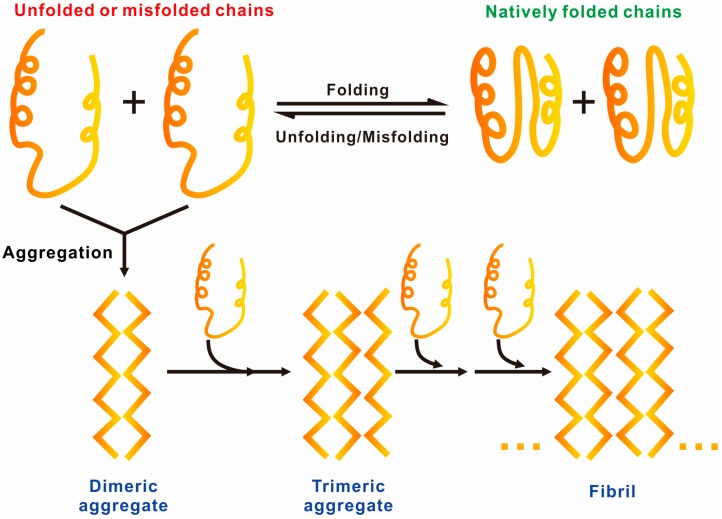
Transformation of proteins from natively forded states to unfolded/misfolded states and the subsequent fibril formation process [10]. Adapted with permission. Copyright 2002, Nature Publishing Group.

Proteins, which consist of one or more long chains of amino acid residues, differ from one another primarily in their amino acid sequence determined by the nucleotide sequence of the corresponding genes [1]. Mainly because of various non-covalent interactions (i.e., hydrogen bonds, ionic interactions, dipole–dipole bonds, etc.) and disulfide bonds between two cysteine residues, proteins will further fold into a specific three-dimensional (3D) structure, which determines their activity and function in living organisms, including replicating DNA, catalyzing metabolic reactions, transporting biomolecules, and responding to stimuli [2,3,4]. This most thermodynamically favorable structure is stabilized depending on the intrinsic properties of the amino acid sequence and multiple contributing influences from the surrounding environment, particularly the crowded cellular milieu in vivo [5,6]. However, the folded protein is prone to alteration induced by amino acid mutation or external forces, such as heating, pH abnormality, or the addition of metal ions, which result in unfolded or misfolded proteins [7,8]. If the unfolded or misfolded proteins cannot be refolded to their normal states or immediately degraded, these proteins may accumulate into three typical aggregates (amorphous aggregates, oligomers, and amyloid fibrils) [9], because of strong stacking between the hydrophobic portions of these proteins in unstable states (Scheme 1).

Protein aggregation is fundamentally associated with a variety of common biological phenomena, such as blood coagulation, invalidation of proteinic drugs, and formation of amyloid plaques [11]. In some cases, protein aggregation plays a positive role in our life. For example, the occurrence of blood coagulation and consequent wound healing relies on the aggregation of a specific protein. The hemostasis process is modulated by a specific enzyme (thrombin), which is capable of activating fibrin proteins from another natural plasma glycoprotein, fibrinogen. Owing to the activity of thrombin, the fibrin proteins first assemble into fibrils and then aggregate into 3D clots for sealing blood outflow. Moreover, the wound is healed by the binding of these fibrils with collagen, which is provided as scaffolds for tissue regeneration [12,13].

However, in most cases, protein aggregation plays a negative role in some undesired biological processes or even serious diseases, such as the formation of cataracts [14,15], sickle cell disease [16], and arterial thrombosis [17]. In addition, the most well-known diseases that are associated with protein aggregation-induced disorders are perhaps the neurodegenerative diseases, including Alzheimer’s disease (AD), Huntington’s disease (HD), Parkinson’s disease (PD), amyotrophic lateral sclerosis (ALS) and prion diseases [18,19,20,21,22]. Taking AD as an example, two main protein aggregates are involved in its pathogenesis: extracellular and intracellular aggregates. The extracellular aggregates, known as neuritic plaques, are constituted with amyloid-*β* (A*β*) peptides. These peptide fragments are derived from the hydrolysis of amyloid precursor protein (APP, a type of membrane protein) catalyzed by cellular secretases. The obtained A*β* peptides first undergo fibrillation aggregation and then plaque formation, which is cytotoxic and may induce apoptosis [23,24]. On the other hand, the intracellular aggregates, known as neurofibrillary tangles, are composed of the microtubule-associated protein tau [25]. Although the pathogenic proteins/peptides of each neurodegenerative disease are specific and individual, all these disorders show similarities in the protein/peptide aggregation process, particularly on the cell membrane. The aggregates are often assembled by fibers consisting of misfolded proteins with a *β*-sheet conformation. On the basis of the aforementioned findings, the research of protein aggregation has shown its great significance for explaining diverse physiological phenomena and helping medical scientists comprehensively understand the pathogenesis of many serious diseases.

### 1.2. General Driving Forces of Protein/Peptide Aggregation

For in-depth research on protein aggregation, we should first understand why and how proteins aggregate. Fortunately, numerous studies have been performed, and various driving forces have been acknowledged by worldwide scientists, which include: (1) van der Waals’ force and hydrophobic interactions between the backbone and side chains; (2) minimizing steric hindrance and maximizing hydrogen bonding; (3) maximizing (minimizing) electrostatic attractions (repulsions); (4) minimizing undesirable interactions between amino acids within the same protein or neighboring ones or between amino acids and the solvent; and (5) maximizing chain entropy [26,27,28]. Because of the aforementioned driving forces, proteins in monomeric state are prone to transform into the aggregated state when their concentration is beyond the limit in solution.

Generally, protein aggregation can be reversible or irreversible. Folded proteins often undergo reversible aggregation and can easily be reversed when diluted 10–100 fold. These aggregated proteins are initially in the folded monomer state and transformed into dimers or oligomers, which are regarded as stable complexes that can coexist with the monomers in dynamic equilibrium or can be separated from the solution. For example, the equilibrium dissociation constant of monomer–dimer (K_d_ = (monomer)^2^(dimer)^−1^) for insulin is 10 μM in the absence of zinc [29]. When the concentration is close to the K_d_ value, the monomer and dimer of insulin will coexist in proximate amounts. By contrast, if the protein solution is diluted to a concentration below the K_d_ value, the monomer will become more stable and hardly be transformed into dimers or oligomers. Besides, the K_d_ value is reported to be closely related to solution pH and temperature [30]. As a matter of fact, proteins can also aggregate into an irreversible network structure, which is not readily disaggregated unless exposed to a solution containing chemical denaturants at an ultrahigh concentration [31,32]. Based on the aforementioned findings, we can presume that the concentration, pH, temperature and specific chemical molecules are the major factors potentially influencing protein aggregation in solution.

### 1.3. Protein Aggregation Modulated by Biological Membranes

As stated above, aggregation in solution often occurs when the concentration of the target protein/peptide is above the K_d_ value. However, A*β* peptides have been shown to aggregate on cell membranes even at picomolar concentrations, which is far less than the critical concentration in vitro [33]. This result implies that the protein aggregation in solution behaves greatly differently from that at the biointerface (i.e., biological membranes) [34]. In fact, the pathological assembly of proteins/peptides into toxic aggregates, which play a critical role in amyloidogenic diseases, usually occurs on membranes. Therefore, researching protein aggregation on membranes seems more significant than that in solution for a comprehensive understanding of these protein aggregates-induced diseases.

**Figure 1:**
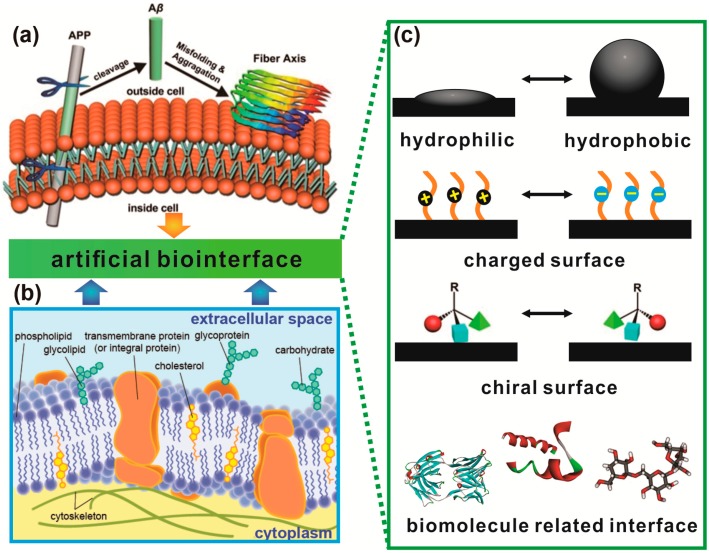
(**a**) Illustration of APP cleavage, A*β* misfolding and oligomer aggregation occurring on cell membranes [41]; (**b**) the cell membranes with complex components (i.e., carbohydrates, lipids, proteins, etc.), structures and various surroundings; and (**c**) several main types of artificial biointerfaces developed from different perspectives to investigate the effects of interfacial properties on protein aggregation. (**a**) Adapted with permission. Copyright 2014, American Chemical Society (ACS).

Again taking AD as an example, the fate of A*β* peptides in amyloidosis critically depends on the properties of cell membranes, which provide the immediate biochemical environment that pathologically alters (shown in Figure 1a,b) [35,36,37,38]. Research indicates that neuronal lipid membranes can effectively enhance the conversion of A*β* peptides into toxic oligomers, which is regarded as a critical phase in AD. Inherited from APP, A*β* peptide naturally possesses the character of amphipathy, provided as an ideal candidate to interact with complex compounds (i.e., carbohydrates, lipids, proteins, etc.) on membranes. Furthermore, APP itself is a typical transmembrane protein whose dimerization in membranes may play a vital role in its cleavage into A*β* peptides [39]. Moreover, evidence suggests that negatively charged lipid components induce dramatic surface accumulation of A*β* peptides, which is driven by electrostatic interactions, and then accelerate their conversion into toxic A*β* aggregates [40].

Except for the offered special biochemical environment, proteins accumulated on membranes are restricted to a 2D space in contrast to the 3D space for proteins accumulated in solution (i.e., enchylema). In the 2D space, the average distance between proteins is related to the square root of the total amount of proteins, while in the 3D space, the average distance is related to the cube root of the total amount of proteins. Hence, the binding of proteins with membranes sharply reduces the average distance between proteins in terms of geometry, which may contribute to the higher protein–protein interactions [42,43]. Moreover, the interaction sides of the proteins (e.g., hydrophobic sides [44]) can be buried in lipid bilayers, accompanying with the confining diffusion of proteins in a 2D space, which may in turn weaken the protein–protein interactions and consequently restrain protein aggregation.

In the past decade, thousands of representative studies have attempted to explain why and how diverse toxic protein aggregates form on membranes. However, the membranes in vivo are too complex to obtain an accurate and direct evidence for their influence on protein aggregation, or particularly amyloidosis. While the role of membranes as promoters of pathological protein aggregation has received preliminary recognition, it has been discerned only for the A*β* peptides in AD and *α*-synuclein in PD. Thus, it is still a tremendous challenge to comprehensively understand the membrane-associated pathological mechanisms of these amyloidogenic diseases. Artificial biointerfaces, which mimic the membranes from the given surface properties, have been regarded as an effective complement for such research, due to their superior flexibility, tenability, and controllability [45,46]. This review presents various artificial biointerfaces, which are divided into four groups according to their special physical, chemical, or biochemical properties (Figure 1c). The modulation of protein aggregation, particularly amyloidosis, by these interfacial properties is discussed as follows (Section 2).

## 2. Hydrophobic–Hydrophilic Interfaces Triggered Accumulation of Amyloid Protein

It is well-known that membrane provides a relatively hydrophobic surface, which constitutes a natural hydrophobic–hydrophilic interface in vivo with cytochylema containing diverse proteins/peptides. On the other hand, the aggregation process of proteins/peptides into amyloid fibrils has been intensively investigated in various in vitro experiments, in which the samples are usually exposed to various hydrophobic substances (e.g., container or stirrer wall, and air) and accumulate at hydrophobic–hydrophilic interfaces, such as solid–water interfaces or air–water interfaces (AWIs) [47]. Therefore, these hydrophobic–hydrophilic interfaces triggered adsorption of proteins and subsequent amyloidosis is of great importance for researches both in in vivo and in vitro.

### 2.1. Effect of the Air–Water Interface on Protein Aggregation

In fact, air is unavoidably introduced into a bulk solution under agitation and shaking. Since air is highly hydrophobic compared to water, the interface between air and the solution generates a denaturing surface on which aggregation has been proven to originate. The natively folded state of proteins is disrupted by the AWI, triggering intermolecular protein attractions [48], which induces a number of unexpected and compelling results. For example, in 2002, Brems et al. reported an inverse relationship between protein concentration and protein aggregation [49]. They monitored the aggregates by using size exclusion high-performance liquid chromatography and found that the protein concentration exerted opposite effects on aggregation, which resulted from quiescent incubation and agitation, respectively. The quiescent treatment of pegylated granulocyte colony stimulating factor (PEG-GCSF, M_w_ = 18,798 Da) showed a result of enhanced aggregation, with a more than 6-fold increase in the strength of the aggregation peak, induced by increasing protein concentration from 1 to 10 mg·mL^−1^. In another similar experiment, the aggregation percentage of pegylated megakaryocyte growth and development factor (PEG-MGDF, M_w_ = 17,432) increased from approximately 7% to 17% over 12 week incubation while the concentration was increased from 0.2 to 2.0 mg·mL^−1^. These results were consistent with the common sense: increasing protein concentration could obviously enhance the collision frequency of proteins and subsequently accelerate the aggregation of nonnative states that originate from the conformational reaction of native states. 

By comparison, opposite results were obtained when the PEG-MGDF solution was increasingly exposed to the AWI through agitation with a vortex mixer. The strength of the aggregation peak successively decreased with increasing protein concentration from 1 to 10 mg·mL^−1^. This unexpected phenomenon could be explained as follows. Folded proteins in bulk solution are commonly amphipathic with the buried hydrophobic groups and the hydrophilic groups exposed to water. The denaturation of proteins could occur at AWI, and partially unfolded proteins contribute to the exposure of hydrophobic groups to the air. Consequently, intermolecular hydrophobic interactions between the exposed hydrophobic groups promote self-association, finally resulting in protein aggregates. Hence, the rate of agitation-induced aggregation is mainly limited by the protein concentration and the surface area of the AWI [50,51]. Brems et al. suggested that the AWI is the rate-limiting factor, and the AWI to protein ratio is the most critical parameter for aggregation. Therefore, if the agitation rate remains constant, the AWI to protein ratio would increase with the decrease in the protein concentration, finally resulting in enhanced aggregation.

**Figure 2:**
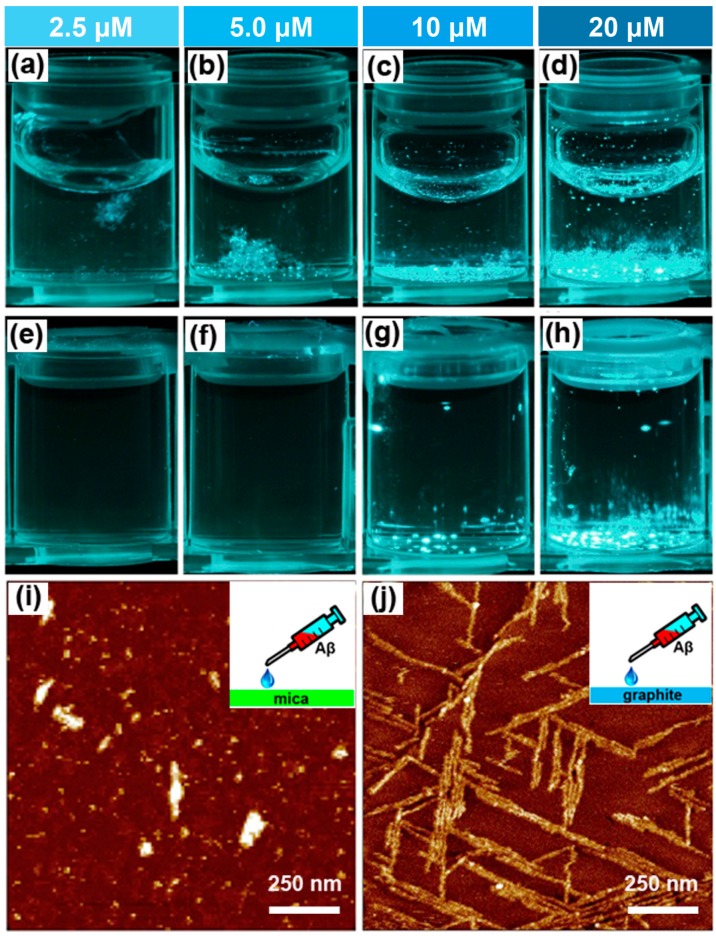
(**a**–**h**) Fluorescence microscopic analysis of A*β*(1–40) aggregation [52]. The reaction mixture containing 2.5 μM (**a**,**e**), 5.0 μM (**b**,**f**), 10 μM (**c**,**g**), or 20 μM (**d**,**h**) A*β*(1–40) was incubated for 22 days at 37 °C without agitation in the presence (**a**–**d**) or absence (**e**–**h**) of the air–water interface (AWI). The sample well was 12 × 9 mm^2^ (height × outer diameter at the bottom). Atomic force microscopy (AFM) images of A*β* aggregates on the hydrophilic mica surface (**i**) and on the highly ordered hydrophobic graphite surface (**j**) [55]. (**a**–**j**) Adapted with permission. (**a**–**h**) Copyright 2010, Elsevier. (**i**,**j**) Copyright 1999, The National Academy of Sciences.

As described in the preceding paragraph, the introduction of the AWI may distinctly accelerate the formation of protein aggregates at a relatively low protein concentration. In organisms, the toxic A*β* aggregates are constituted by misfolded A*β* peptides (e.g., A*β*(1–40) and A*β*(1–42)) of ultralow abundance at a similar AWI. In 2010, the critical role of AWI and agitation in the nucleation process of A*β*-fibrils was clearly revealed by Naiki et al. [52]. They examined the aggregation kinetics (22 days) of A*β*(1–40) at different concentrations ranging from 2.5 to 20 μM, with the presence or absence of AWI. The aggregates were directly monitored by fluorescence spectroscopy or fluorescence microscopy with assistance of thioflavin T (ThT, a specific amyloid dye). When AWI was introduced, A*β* fibril formation was clearly observed in all the solutions at different concentrations. By contrast, without the introduction of AWI, A*β* fibril formation was observed only when the concentration increased to 10 μM or even higher (Figure 2a–d). More importantly, when the AWI was introduced into the A*β* solution at a lower concentration (i.e., 5 μM), the ThT-reactive aggregates (short fibrils) were first observed at the AWI after 10 days incubation, whereas no fibril formation was observed in bulk solution. Furthermore, with agitation by vortexing or rotating, the aggregation process in this A*β* solution was considerably accelerated; short fibrils were also first observed at the AWI before being observed in bulk solution through mixture and diffusion. It was widely accepted that A*β* aggregates are generated by a nucleation-dependent polymerization mechanism, which is composed of two steps: the nucleation of A*β* peptides into short fibrils and the extension of the fibril ends by attachment with monomers [53]. In another work reported by Nagaraj et al., it has been shown that the AWI also influences the aggregation of shorter amyloidogenic peptides through enhanced self-assembly [54]. These works provide evidence that the AWI plays an essential role in the formation of A*β*-fibrils at a low concentration, in turn suggesting that the interference from the AWI should be carefully considered for a more precise evaluation of protein amyloidosis modulated by biological interfaces in vitro.

Luckily, over the past decade, much effort has been made to eliminate the effects of AWI, protecting proteins in a direct or indirect way [56,57,58]. In 2013, Nidetzky et al. successfully prevented the aggregation of recombinant human growth hormone (rhGH) at the AWI by adding surface-active compounds, namely Pluronic F-68 (M_w_ = 8400 Da), which is a well-known triblock copolymer consisting of two hydrophilic polyethylenoxide (PEO) side chains and a hydrophobic polypropylene glycol (PPG) in the center [59]. They added Pluronic F-68 at an optimal concentration of approximately 240 μM, which was below its own critical micelle concentration [60] and slightly exceeded the adopted protein concentration (150 μM). The added Pluronic F-68 not only suppressed foam formation similar to PPG 2000, but also completely inhibited rhGH aggregation. Conformational analysis through small-angle X-ray scattering (SAXS) and circular dichroism (CD) spectroscopy showed that in the presence of Pluronic F-68, the soluble rhGH retained its native folded state, featuring original secondary structural elements under both stirring and aeration. Nidetzky et al. proposed that this phenomenon was attributed to the direct hydrophobic interaction between Pluronic F-68 and the AWI, which prevented rhGH attachment to the AWI, finally resulting in the inhibition of rhGH aggregation by Pluronic F-68. Moreover, AWI-induced aggregation of rhGH was suggested to include desorption of nonnative proteins from the AWI as a critical step. The modulation of this process may be a promising strategy to avoid AWI-induced aggregation.

### 2.2. Effect of the Hydrophobic Surface on Protein Aggregation

As we know, Teflon surfaces may be the most typical mimics of the hydrophobic nonpolar lipid membrane surface. In 2003, Giacomelli et al. reported the effect of hydrophobic Teflon particles on the secondary structure of A*β*(1–40) [61]. Although both A*β*(1–40) and Teflon carried a negative charge at physiological pH, Teflon particles induced substantial adsorption of A*β* from the bulk solution (pH = 7), which resulted from hydrophobic interaction between Teflon and A*β*(1–40). The adsorption was accompanied with the conformational conversion of A*β* from *β*-sheet structures into *α*-helical structures. Notably, as the Teflon surface became crowded with successive adsorption of A*β*, intermolecular interactions between the adsorbed A*β* peptides induced conformational transitions back into *β*-sheet structures. Comparable results were obtained in a previous study by Schladitz et al., wherein monolayer of A*β*(1–40) aggregates arranging in an antiparallel *β*-sheet structure was observed at the AWI [62]. Meanwhile, similar results were obtained with other hydrophobins, such as insulin [63]. The reasons why the absorbed hydrophobins present distinct secondary structures at these two hydrophobic–hydrophilic interfaces (Teflon surfaces and AWI) is unclear and remain to be revealed. One explanation is that the α-helical structure is trapped by the Teflon surfaces, whereas it is spontaneously and rapidly converted into the *β*-sheet structure (more stabilized conformation) at the AWI. Moreover, the electrostatic interactions also play a crucial role in stabilizing the helical state and preventing the conversion of A*β* into the *β*-sheet structure, as suggested by Marcinowski et al. [64].

Early in 1999, Kowalewski and Holtzman proposed that surface wettability influences the aggregation of A*β*(1–42) [55]. In order to observe the amyloid formation process directly on hydrophobic graphite and hydrophilic mica, they first utilized in situ atomic force microscopy (AFM), which can be operated in the liquid phase and track the time-dependent growth of individual aggregates on the surface (Figure 2e,f). Notably, substrate-templated self-assembly of A*β* was observed on the hydrophobic graphite; uniform and elongated sheets of A*β* were oriented along three directions at 120° to each other, resembling the crystallographic symmetry of the graphite surface. This result implies that the formation of *β*-sheets might be dominated by nonlocal intermolecular interactions, with resemblance to the crystallization of linear macromolecules, which greatly differs from that of helical structures in bulk solution. In contrast, at the surface of hydrophilic mica, A*β*(1–42) formed particulate, pseudo-micellar oligomeric assemblies, which tended to form long fibers at the higher A*β* concentration (increased from 10 to 500 μM). In 2000, Blackley et al. obtained similar results for A*β*(1–40) aggregation on mica [65].

**Figure 3:**
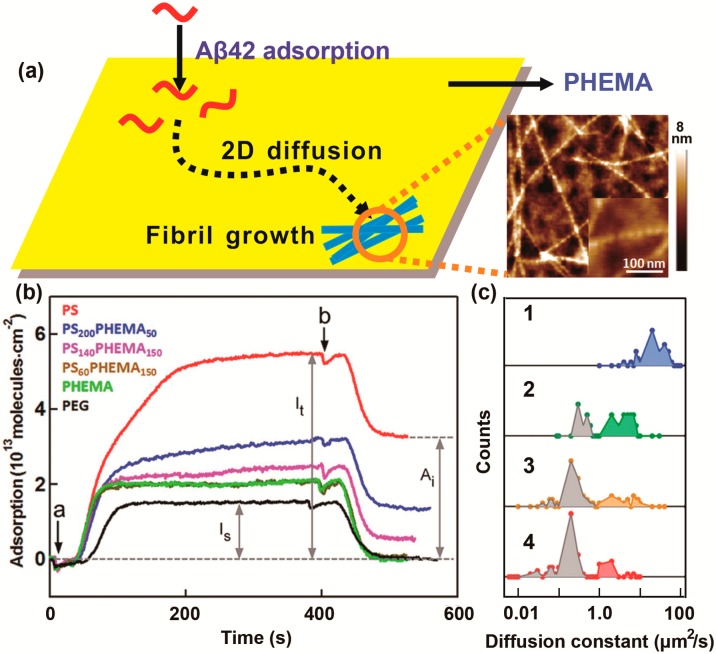
(**a**) Illustration of A*β*42 fibril formation and the AFM image of poly(2-hydroxyethyl methacrylate) (PHEMA) surface after incubation in the A*β*42 peptide solution (1 μM in phosphate buffered saline (PBS)) at 37 °C for 18 h; (**b**) surface plasmon resonance (SPR) responses for the A*β*42 peptide adsorption on the poly(ethylene glycol) (PEG), PHEMA, polystyrene (PS)_60_-*b*-PHEMA_150_, PS_140_-*b*-PHEMA_150_, PS_200_-bPHEMA_50_, and PS surfaces (immersed in a 1 μM A*β*42 solution in PBS for 10 min); and (**c**) histograms of the diffusion coefficients of A*β*42 monomers on four polymer films: (1) PHEMA; (2) PS_140_-*b*-PHEMA_150_; (3) PS_200_-*b*-PHEMA_50_; and (4) PS. The highest growth extent and the longest fibrils were observed on the PHEMA surface, where the highest diffusion constant (D = 22 ± 12 μm^2^·s^−1^) was obtained for the reversibly adsorbed A*β*42 peptides [66]. (**a**–**c**) Adapted with permission. Copyright 2012, ACS.

Although increasing evidence has demonstrated that the hydrophobic interface is crucial in the adsorption of hydrophobins, Zhu et al. proposed the critical role of the diffusion constant of the adsorbed peptide in subsequent self-assembly (Figure 3a) [66]. They prepared gold or glass surfaces with various θ_C_ values (static water contact angle) by using diverse polymer coatings that ranged from hydrophilic poly(ethylene glycol) (PEG, θ_C_ ≤ 10°) to intermediate poly(2-hydroxyethyl methacrylate) (PHEMA, θ_C_ = 55° ± 2°) and hydrophobic polystyrene (PS, θ_C_ = 102° ± 2°). The interaction of these surfaces with A*β*42 was first probed using surface plasmon resonance (SPR) spectroscopy (Figure 3b). On the hydrophilic surface (PEG) or certain intermediate surfaces (PHEMA and PS_60_-*b*-PHEMA_150_), the amount of irreversibly adsorbed peptides (A_i_) was negligible. However, this amount increased to (0.5 ± 0.1), (1.3 ± 0.2), and (3.2 ± 0.1) × 10^13^ molecules·cm^−2^ on the PS_140_-*b*-PHEMA_150_, PS_200_-*b*-PHEMA_50_, and PS surfaces, which is directly correlated with hydrophobicity. The amount of reversibly adsorbed peptides (A_r_) increased from (0.5 ± 0.1) × 10^13^ molecules·cm^−2^ on PHEMA to (0.8 ± 0.1) × 10^13^ molecules·cm^−2^ on PS, while the A_r_ value was negligible on PEG.

Furthermore, to evaluate the mobility of these absorbed peptides on diverse surfaces, single-molecule fluorescence tracking experiments were performed by utilizing total internal reflection fluorescence microscopy (TIRFM). Subsequently, the diffusion coefficient constants (D) were calculated from the time-dependence trajectories (Figure 3c) [67]. The combined AFM images of polymer-coated thin films incubated in A*β*42 solution (1 μM in phosphate buffered saline (PBS), 37 °C) for 18 h suggested that growth of fibril-like A*β* aggregates on the surface required sufficient amounts of reversibly adsorbed and mobile peptide molecules, which presents a negative correlation with the A_i_ value. This finding was confirmed by the observation of the highest growth extent and the longest fibrils on the PHEMA surface, where there was a sufficient amount of reversibly adsorbed peptides with the highest mobility (D = 22 ± 12 μm^2^·s^−1^). The growth extent of fibrils decreased with the decrease in peptide mobility or increase in A_i_ on PS_60_-*b*-PHEMA_150_ and PS_140_-*b*-PHEMA_150_ and was negligible on the PS_200_-*b*-PHEMA_50_ or PS surface, on which most adsorbed peptides were firmly anchored and had poor mobility. On the hydrophilic PEG surface, no A*β*42 peptides were absorbed, and consequently no fibril growth was observed. According to the aforementioned results, the aggregation of A*β*42 is highly sensitive to surface wettability, which mainly influences the adsorption and subsequently diffusion of A*β*42 peptides at the 2D surface. Further mechanism was proposed that fibril growth is induced by the attachment of the A*β* molecule absorbed on the solid surface to the end of a short fibril, rather than the A*β* molecule in bulk solution. As a matter of fact, early in 2006, Miranker et al. [68] suggested that the peptides capable of free diffusion on cell membrane surfaces are responsible for the membrane-mediated fibrillation of human islet amyloid polypeptide, which is consistent with this study by Zhu et al. These studies inspire us to investigate the controllable aggregation behaviors of hydrophobins at the smart surfaces that can be switchable between superhydrophobic state and superhydrophilic state. We believe that such intelligent mode in the studies of interface-triggered protein aggregation may attract more attention in the near future, for the purpose of mimicking the subtle and fast-changing environment provided by cells in vivo.

### 2.3. Dominated Effect of Hydrophobic–Hydrophilic Interface on Protein Aggregation in the Presence of Metal Ions

It has been widely accepted that metal ions in vivo, particularly Cu^2+^ and Zn^2+^, interfere with protein amyloidosis in neurodegenerative diseases such as PD or AD [69,70,71,72]. The effects of these metal ions range from acceleration to inhibition with different coordination affinities, depending on binding properties. For example, complexation of Zn^2+^ often has a tendency to accelerate aggregation [73], while the effects of Cu^2+^ on aggregation may be either acceleration or inhibition, depending on solution conditions [74]. In 2011, Hoernke and Brezesinski demonstrated amyloid formation at hydrophobic–hydrophilic interfaces with the chelate effect of Cu^2+^ and Zn^2+^, by using designed peptides carrying specific metal-ion binding sites (i.e., histidine residues, His) [75]. These binding sites were located in the residue i and four amino acids further on peptide (i, i + 4) or in residues i and i + 2, respectively. The data of CD showed both peptides preserved their native disordered state in bulk solution with addition of Cu^2+^ or Zn^2+^, implicating that the metal ions exerted no influence at the lower peptide concentration. However, at higher concentrations, the chelation of both Zn^2+^ and Cu^2+^ with peptide (i, i + 4) stabilized the *α*-helical conformation, while for peptide (i, i + 2), Cu^2+^ stabilized the *α*-helix but Zn^2+^ accelerated the formation of *β*-sheets.

Moreover, the peptides absorbed at the hydrophobic–hydrophilic interface were forced to immediately rearrange into α-helix (a low-energy conformation), having poor relationship with the peptide concentration [76]. Peptide (i, i + 4) in the α-helical conformation could still provide a chelate ligand because of two close His residues and finally be stabilized at interfaces by metal ions. By contrast, the His of i and i + 2 was rearranged on opposite sides of the helix, inhibiting chelation with Zn^2+^, whose binding affinity was orders of magnitude lower than that of Cu^2+^ [77]. The chelate effect of Zn^2+^ only arose in the case of *β*-sheet formation, due to the presence of close His binding sites. Hence, for peptide (i, i + 2), Zn^2+^ could not inhibit the conformational transition from α-helix into *β*-sheets by stabilizing the *α*-helical structure through complexation. These results are consistent with those of Brezesinski et al. in 2012 [78]. They concluded that the hydrophobic–hydrophilic interface largely determines the conformational transition of the absorbed peptide and mainly influences peptide aggregation, compared with metal ions or even peptide concentration. This is because at the interface, metal-ion complexation only slightly affects the secondary structures of absorbed peptides, which are restricted to a 2D space.

## 3. Protein Adsorption and Aggregation Influenced by Surface Charge

**Figure 4:**
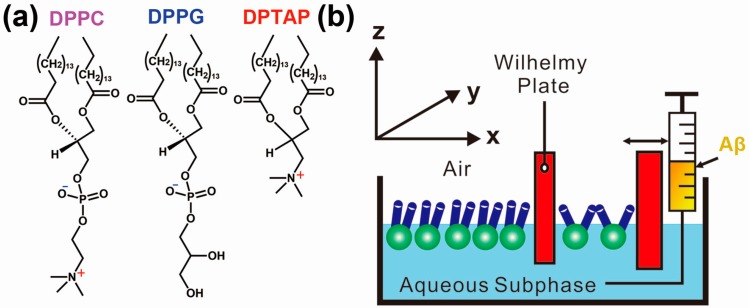
(**a**) Structures of the lipids used. The lipids have identical saturated 16-carbon acyl chains but differ in their head group architecture and charge; (**b**) Schematic of a Langmuir trough containing a lipid monolayer. The movable barrier controls the lipid density of the film and the Wilhelmy plate measures the surface pressure. A*β*40 solution (250 nM) was injected into the aqueous subphase of the trough using an l-shaped syringe underneath the barrier [84]. (**a**,**b**) Adapted with permission. Copyright 2008, WILEY-LISS.

Recently, increasing evidence has demonstrated the effect of the charged lipid membrane on the formation of A*β* fibrils [79,80]. In contrast to zwitterionic lipids, anionic lipids containing a phosphate group on the head group have been proven to enhance A*β*’ association or insertion into membranes [81,82] through electrostatic interactions, which subsequently induce *β*-sheet formation and consequently promote fibrillogenesis [83]. To make a better understanding of the molecular-level details, Lee et al. used in situ X-ray diffraction (reflectivity) and neutron reflectivity to observe the adsorption and aggregation of A*β*40 on diverse lipid monolayers composed of the cationic lipid 1,2-dipalmitoyl-3-trimethylammonium propane (DPTAP), the anionic lipid 1,2-dipalmitoylsn-glycero-3-(phospho-rac-(1-glycerol)) (DPPG), or the zwitterionic lipid 1,2-dipalmitoyl-sn-glycero-3-phosphocholine (DPPC) at AWI (shown in Figure 4a) [84]. Firstly, a temperature-controlled Langmuir trough, which was equipped with a Teflon barrier for changing the surface area and a Wilhelmy plate balance for measuring the surface pressure (p), was mounted on the diffractometer. After calibration of the pressure sensor at 30 °C, 240 mL of aqueous subphase was decanted into the trough. Then DPPG dissolved in chloroform containing 10% methanol, and DPPC and DPTAP dissolved in chloroform were respectively spread at the air–water interface. This system was allowed to equilibrate for 15 min to ensure the complete evaporation of the organic solvent. After that, the lipid monolayer was compressed to 23 or 30 mN/m and the desired pressure was kept constant via a feedback loop. Finally, A*β*40 solution was injected into the subphase of the trough using an l-shaped syringe underneath the barrier without disturbing the lipid film. The X-ray and neutron scattering data indicated the folding and aggregation of A*β*40 occurred at the submicromolar concentration range (250 nM) in a short time of 1 h. By introducing the DPPG–water interface, the intensity of the peak of A*β* ordered in *β*-sheets increased compared with that obtained from A*β* peptides absorbed at the bare AWI. The much narrower FWHM (peak width at half height) of the Bragg peak of A*β* binding with DPPG implies the A*β* aggregates are more than two times larger than those observed at the bare AWI. Moreover, the associated A*β* layer exhibited diverse total thickness depending on different lipids described previously. The associated A*β* multilayers were induced by the monolayer composed of DPPG on water with a thickness of approximately 4 nm, and for DPPC and DPTAP monolayers, thickness ranged from 0.73 to 1.27 nm. This unique template role of anionic DPPG monolayer in A*β*40 assembly was mainly ascribed to the attractive dipole–charge interaction between A*β*40 and DPPG, because the charge on A*β*40 was estimated to be 0.2 in water (pH 5.5) by Protein Calculator v3.3 (The Scripps Research Institute). In addition, they also showed the A*β* association-induced disruption of lipid packing in the membrane, which is implied as a possible toxicity pathway. Meanwhile, Majewski and Chi observed similar full-length tau (hTau40) aggregates at these three lipid membranes respectively composed of the cationic DPTAP, anionic DPPG and zwitterionic DPPC in 2012 [85]. It is mentionable that hTau40 is highly surface active, selectively inserts into the anionic DMPG lipid monolayers and induces structural compaction of tau proteins and membrane disruption, indicating possible membrane-based mechanisms of tau aggregation and toxicity in neurodegenerative diseases [86,87,88].

**Figure 5:**
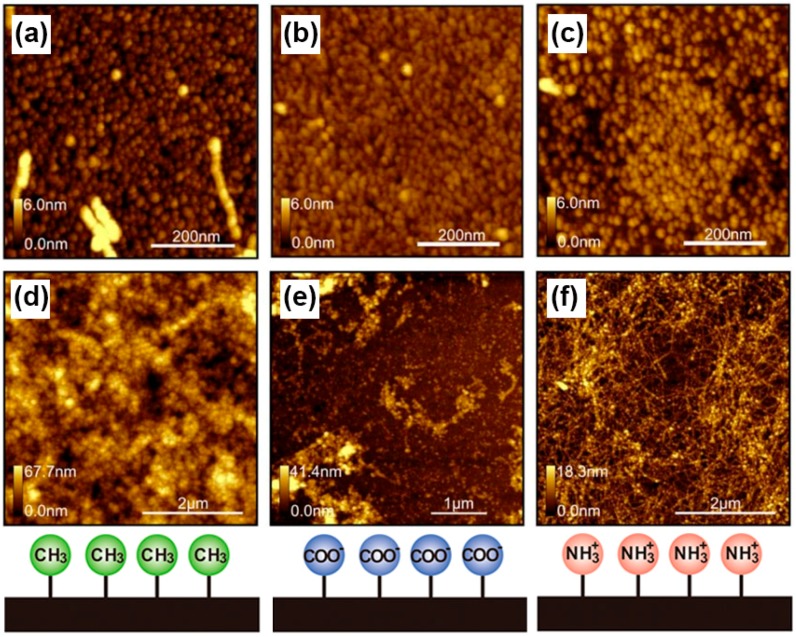
High-resolution images of AFM topography of A*β* aggregates formed on modified surfaces: (**a**) CH_3_-modified; (**b**) COOH-modified; and (**c**) NH_2_-modified surfaces, after incubation in A*β*(1–42) solution (500 mg·mL^−1^) for 1 h at 37 °C. AFM topography images (5 × 5 μm^2^) of the amyloid fibrils formed on: (**d**) CH_3_-modified; (**e**) NH_2_-modified; and (**f**) COOH-modified surfaces, after incubation in A*β*(1–42) solution (500 mg·mL^−1^) for 22 h at 37 °C. These data indicate the first monolayer formed was composed of densely packed oligomers on each of the three surfaces. However, after 21 h of incubation, vimineous fibrils were observed on the NH_2_- and COOH-terminated surfaces whereas only amorphous globular clusters were found on the CH_3_-terminated surface [89]. (**a**–**f**) Adapted with permission. Copyright 2008, Public Library of Science.

In 2011, simplified models of charged surfaces were designed by Leonenko et al. to investigate their effect on amyloid fibril formation [89]. They used high-resolution AFM to study the accumulation and aggregation of A*β*(1–42) (pH 7.8, at 37 °C) on surfaces terminating with different chemical groups, including positively charged –NH_2_, negatively charged –COOH, and hydrophobic –CH_3_. AFM images showed the first monolayer formed was composed of densely packed oligomers on each of these charged surfaces, which were incubated in A*β* solution for 1 h (Figure 5a–c). These small building blocks were spherical at the CH_3_-terminated surface and triangular both at the negatively charged COOH- and positively charged NH_2_-modified surfaces, but were more tightly packed at the NH_2_-modified surfaces. This finding implies the possible effect of electrostatic interactions on oligomer folding and assembly and the consequential small aggregates. As proposed by Wang et al. [90], A*β* can move more freely at the CH_3_-modified surface and stick to COOH- and NH_2_-modified surfaces, inducing more ordered A*β* deposits. The AFM images demonstrated that visible larger aggregates were observed at each of these surfaces after 21 h (Figure 5d–f). The A*β* aggregates on the hydrophobic CH_3_-modified surface were in the form of amorphous globular clusters with a mixture of various sizes, but no vimineous fibrils were observed. However, for the hydrophilic surfaces, both the NH_2_- and COOH-terminated surfaces were covered with combination of fibril-like aggregates and uniformly sized globular clusters (approximately 25 nm in diameter). Almost the entire CH_3_-modified surface (94%) was covered with the second layer composed of amorphous globular A*β* clusters, while for the NH_2_-modified surface, the coverage distinctly decreased to 23.7%. Hence, fibril-like aggregates with a high coverage (81.3%) were only observed on the negatively charged COOH-modified surface, indicating the critical role of electrostatic attraction in the elongation phase.

Furthermore, the nonlinear Poisson–Boltzmann equation (PBE) has been utilized to analyze the electrostatic interactions between amyloid deposits and modified surfaces, as an effective complement for AFM data. Difference in surface charge distribution has been determined for A*β* monomers, dimers, or much larger oligomers. The oligomers ordered as *β*-sheets show higher collective polarity and induce stronger electrostatic interaction with charged modified surfaces. This interaction is regarded as the possible driving force for the formation of more amyloid fibrils at charged surfaces, compared to CH_3_-modified surfaces. However, surface-induced electrostatic force can also cause electrostatic potential redistribution in the monomers near the surfaces and in turn lead to the conformational transition of peptides, subsequently inducing electrostatically driven fibrillogenesis [91].

The results obtained under neutral conditions are consistent with those of McMasters et al. [92], who used reflection-absorption infrared spectroscopy and scanning force microscopy (SFM) to investigate the aggregation of A*β*(10–35) on *ω*-substituted alkanethiol-modified gold carrying positively or negatively charged, or hydrophobic functional groups (i.e., COOH, OH, SO_3_H, or CF_3_) at pH 11.5. The CF_3_ monolayer showed the similar result of the formation of amorphous aggregates with *α*-helical structures and without the formation of fibril-like A*β* deposits, which correlated with the CH_3_-modified surface-induced aggregation of A*β* demonstrated previously.

In contrast to these short-range noncovalent (e.g., hydrophobic) interactions, electrostatic forces between the protein and surface are long-range interactions and can be transformed from attractive into repulsive forces under altered conditions of the bulk solution. In 2015, Schwartz et al. [93] utilized single-molecule TIRFM (SM-TIRFM) to investigate the effects of pH and ionic strength on the electrostatically driven adsorption and desorption of bovine serum albumin (BSA) at the silica–water interface. The steady-state adsorption rate coefficients (*Kads*) calculated from SM-TIRFM was used to represent the absolute adsorption rate. At pH below 4.7 (the isoelectric point (pI) of BSA), where electrostatic force between the positively charged BSA and negatively charged silica surface was attractive, fast adsorption was observed with a *Kads* exceeding 1000 nm·s^−1^. By contrast, at the pH above 4.7 (i.e., 7.4), the *Kads* sharply decreased to approximately 2.9 nm·s^−1^ because of electrostatic repulsion between the negatively charged protein and surface. With the addition of 100 mM NaCl into this solution at pH 7.4, a 1000-fold increased *Kads* of approximately 2000 nm·s^−1^ was obtained. However, for the solution at pH 2.6, the *Kads* remained similar to that measured under low ionic strength conditions. These findings suggest that the energy barriers of adsorption kinetics associated with electrostatic interactions can be modulated by the pH and ionic strength, which provides potential means of stabilizing proteins and preventing their aggregation.

## 4. Protein Aggregation Modulated by Chiral Interface

**Figure 6:**
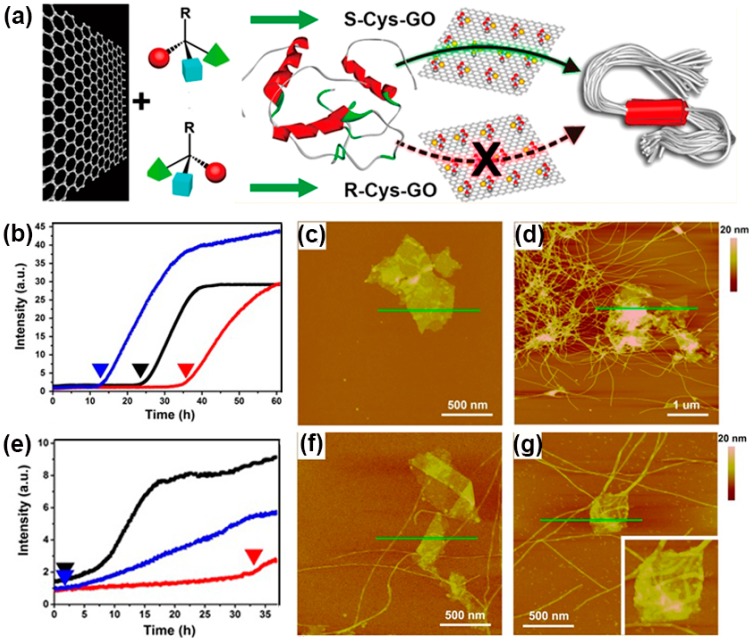
(**a**) Illustration of the R-Cys-graphene oxide (GO) and S-Cys-GO films and their influence on A*β*(1–40) aggregation. Time-dependent fluorescence intensity changes: (**b**) R- (red) and S-Cys-GO (blue) added at the beginning of incubation; (**e**) R- (red) and S-Cys-GO (blue) added after 10 h of incubation. Black curves are all the control experiment with only A*β*(1–40) peptides. All the experiments were performed in the working solutions (100 µL) containing 25 µM A*β*(1–40) peptides, 25 µM ThT in phosphate buffer solution (50 mM, pH 7.4), and then 100 µL aqueous solution of various chiral GO samples (0.15 mg·mL^‒1^) were added to the pre-prepared solutions, respectively. AFM images of R(S)-Cys-GO after incubation in A*β*(1–40) solutions (25 µM): (**c**,**f**) R-Cys-GO; (**d**,**g**) S-Cys-GO; and (**c**,**d**) R(S)-Cys-GO were added at the beginning of incubation; and (**f**,**g**) GO samples were added after 10 h of incubation at 37 °C [41]. (**a**–**g**) Adapted with permission. Copyright 2014, ACS.

Cytomembranes are mainly composed of phospholipid bilayers with a highly ordered arrangement of phospholipid molecules, which possess chirality and exhibit a high preference to l-enantiomers. From the insights of bionics, many compelling studies have shown the effect of chirality on the protein adsorption process [94,95,96] and even on cell behaviors [97,98]. In 2014, Sun et al. showed that surface chirality might strongly influence the aggregation of A*β*(1–40) (Figure 6a) [41]. Utilizing cysteine (Cys) enantiomer-modified graphene oxide (GO) as a model platform, they monitored the amyloid formation process through ThT fluorescence spectroscopy and AFM. The formation of A*β*(1–40) fibrils was previously reported to mainly involve nucleation and elongation phases [99]. Hence, to investigate these processes, two groups of experiments were performed respectively with various GO samples (0.15 mg·mL^−1^) added into A*β*(1–40) solutions (25 μM, 37 °C) at different time points. In the first group, GO samples were added at the beginning of incubation to study the effect on the nucleation phases. The time dependence of fluorescence intensity changes (Figure 6b) showed that (1) aggregation of A*β*(1–40) occurred after 24 h, and fibrillation formation was completed after 38 h; (2) the addition of R-Cys-GO probably inhibited the nucleation phase with postponing it for nearly 11 h; and (3) the addition of S-Cys-GO exerted an opposite effect and preponed this process by approximately 12 h. AFM results provide visual evidence for the inhibitory effect of R-Cys-GO (Figure 6c,d). Interlinked fibrils were found at the S-Cys-GO surfaces, with the maximum height increased by 30 nm, whereas the R-Cys-GO surface was rather clean and without any long fibrils.

To study their effect on the elongation phase, Cys-modified GO samples and ThT were added at the 10th hour when the oligomers had formed and elongation began. The growth curves (Figure 6e) showed that after R-Cys-GO addition, no obvious increase in fluorescence intensity occurred until the 32nd hour; thereafter, the increase was relatively slight. By contrast, the fluorescence intensity increased considerably and immediately after the addition of S-Cys-GO samples. These results indicated the suppression of A*β*(1–40) elongation phase by R-Cys-GO, which was also confirmed using AFM, as illustrated in Figure 6f,g.

This study provides new insight into the influence of the surface on protein aggregation (i.e., formation of amyloid aggregates). The stereoselective interaction-induced chiral effect was suggested inducing visibly different nucleation and elongation processes. Furthermore, when a long-chain spacer group was introduced in another control experiment to increase the distance between the chiral site and the GO surface, the aforementioned chirality effect of R-DCys-GO or S-DCys-GO was not as strong for amyloid formation compared with that of R-Cys-GO or S-Cys-GO samples. This result indicates the critical role of a sufficiently short distance in the synergy between chiral interaction and the surface.

**Figure 7:**
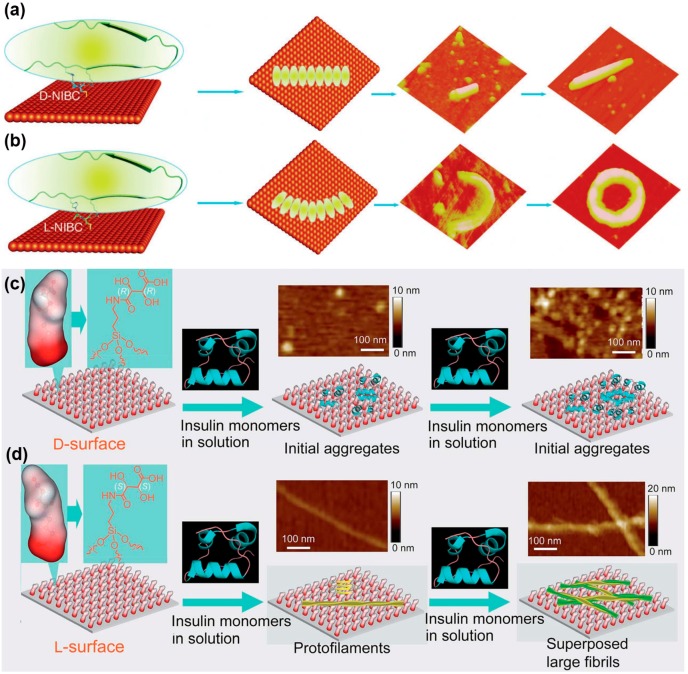
The different possible alignment modes of the *β*-hairpin that are guided by d- (**a**) or l- (**b**) *N*-isobutyryl cysteine (NIBC)-modified surfaces through stereoselective recognition enhanced by electrostatic interactions. A*β*(1–40) peptides at an ultralow concentration (1 μM) have a tendency to form ring-like aggregates on the l-NIBC-modified surface and rod-like aggregates on the d-NIBC-modified gold substrates [100]. Schematic illustration of the different aggregation behaviors of insulin at the d- (**c**) and l- (**d**) tartaric acid-modified surfaces. Insulin at a high concentration of 10 μg·mL^−1^ tends to form unbranched long fibrils on the l-surface, whereas the d-surface was only covered with a monolayer of initial aggregates and no fibril-like aggregates were found [102]. (**a**–**d**) Adapted with permission. (**a**,**b**) Copyright 2014, Wiley Online Library. (**c**,**d**) Copyright 2014, Royal Society of Chemistry (RSC).

In 2014, Sun et al. reported another appealing work in this field. They proposed a surface chirality-induced two-step stereoselective assembly process for A*β*(1–40), which consisted of electrostatic interaction-enhanced adsorption and subsequent stereoselective recognition [100]. *N*-isobutyryl cysteine (NIBC)-enantiomer-modified gold substrates were immersed in a A*β*(1–40) solution with an ultralow concentration (1 μM) at 37 °C for 12 h, and the aggregates were observed using AFM. As illustrated in Figure 7a,b, A*β*(1–40) tended to form ring-like aggregates (approximately 140 nm in outside diameter) on the l-NIBC-modified surface and rod-like aggregates (approximately 40 nm in width) on the d-NIBC-modified gold substrates. The different average Young’s modulus of ring-like aggregates (239 ± 34 MPa) and rod-like aggregates (281 ± 38 MPa) implied the distinct arrangement of these two aggregates, which was inferred to be caused by stereospecific interactions between A*β*(1–40) and D(L)-NIBC moieties.

On the other hand, AFM-controlled tip-enhanced Raman scattering (AFM-TERS), a near-field technique combining the high spatial resolution of scanning probe microscopy and the chemical information provided by Raman spectroscopy [101], was utilized for in situ conformational analysis of these two A*β* aggregates. The data given by the amide I region in the Raman spectrum showed that the ring-like and rod-like aggregates possessed similar secondary structures and assembled as *β*-hairpins with the existence of *β*-turn and *β*-sheet. However, compared with mature fibrils, much stronger *α*-helix and random coil signals were obtained for these aggregates, implying the formation of well-ordered *β*-sheets were not yet complete in both aggregates, as observed using AFM.

Further research was performed by splitting A*β*(1–40) into eight pentapeptides, and their association constant with D(L)-NIBC was determined through the fluorescent titration experiments at physiological conditions (pH of approximately 7.4). Combining these results with the subsequent site-specific replacement experiments, possible electrostatic interaction sites (R5 and K16) and the chiral interaction site (H14) of A*β*(1–40) were proposed on the basis of numerous previous studies [103,104].

Both the studies by Sun et al. contribute to understanding of the aggregation mechanism of A*β* peptides on cell membranes from the perspective of surface chirality, which may provide new insight into the therapeutics of neurodegenerative diseases. More interestingly, a similar chiral effect was not only obtained for A*β* aggregation but also for the insulin assemblies by Qu et al. [102]. In their study, classical biogenic chiral molecules, d- and l-tartaric acid, were assembled into monolayers on mica substrates, constructed as a model chiral surface. As shown in Figure 7c,d, both the d- and l-surfaces were partially covered by the adsorbed insulin after incubation with a low concentration of insulin solution (0.1 μg·mL^−1^) for 24 h. When the insulin concentration was increased to 10 μg·mL^−1^, the d-surface was fully covered with a monolayer of initial aggregates of insulin (most aggregates were approximately 5 nm in size), which were similar to the aggregates observed at the low concentration. By comparison, the higher concentration of insulin induced multilayered, unbranched long fibrils on the l-surface. A possible explanation is that with increasing insulin concentration, the relatively higher interaction between the d-surface and insulin maintains the structure of insulin, because of the high energy barrier for fibril nucleation. However, for the l-surface, the moderate binding force between the l-surface and insulin enables the accumulation of insulin monomers on the l-surface, forming protofilaments and finally superposed long fibrils. Moreover, Qu et al. demonstrated the contrasting cellular effect of insulin accumulation on these two surfaces on neuronal PC12 cells. Fluorescence microscopy images showed a high proliferation and differentiation rate for PC12 cells on the insulin-accumulated d-surface, whereas neither proliferation nor differentiation was observed for PC12 cells on the insulin-accumulated l-surface. This result may be induced by the insulin retaining its bioactivity on the d-surface, whereas the insulin fibrils did not exhibit similar bioactivity on the l-surface. Through this study, the surficial chiral effect was successfully transferred into opposing cellular behaviors. As protein adsorption at diverse interfaces is ubiquitous for tissue engineering and cell adhesion, the new insights into surface chirality may facilitate the engineering of novel biomaterials.

## 5. Protein Aggregation Modulated by Biomolecules and Their Interfaces

**Figure 8:**
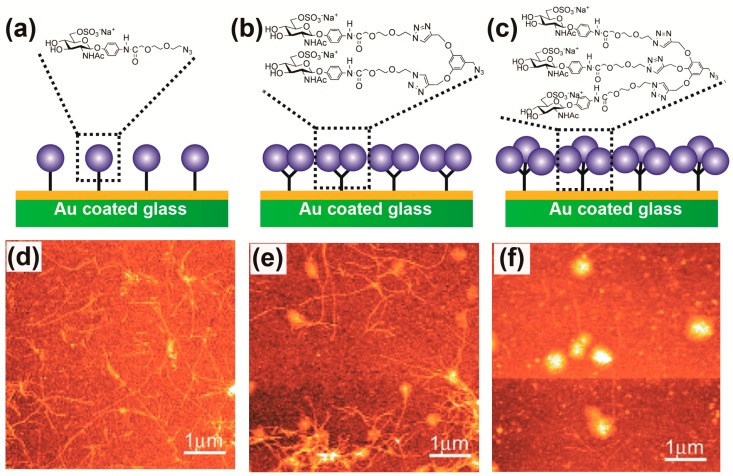
Illustrations of the mono- (**a**), di- (**b**) and tri- (**c**) valent 6-sulfo-*N*-acetyl-d-glucosamine modified glass substrates with gold coating. Morphology observed by AFM on the monovalent (**d**) divalent and (**e**) trivalent (**f**) glycoclusters modified surfaces after they were incubated in a A*β*(1–42) solution (10 μM) for 12 h at 25 °C. These results indicate that A*β*(1–42) tends to form fibril-like aggregates on the monovalent glycocluster-modified surface, while on the multivalent glycocluster-modified surface, A*β*(1–42) tends to form globular aggregates [109]. (**a**–**f**) Adapted with permission. Copyright 2010, ACS.

Various saccharides on the cell membrane have been reported to contribute to the amyloid formation of proteins via carbohydrate–peptide interactions [105,106]. Particularly, the specific binding of A*β* [107] or *α*-synuclein [108] to glycosaminoglycans (i.e., heparin and ganglioside clusters) is closely associated with amyloidosis. In 2010, Miura et al. investigated the effect of glycoclusters on the aggregation behavior of A*β* by preparing various self-assembled monolayers consisting of multivalent glycoclusters on a gold substrate through the method of click chemistry [109]. In this work, mono-, di-, and tri-valent 6-sulfo-*N*-acetyl-d-glucosamine (6S-GlcNAc) (denoted as **G1**, **G2**, and **G3**, respectively) were chosen as representative glycoclusters (Figure 8a–c), because they are the most abundant structure of heparin and these 6S-GlcNAc-modified surfaces may lead to a mimicking result in vivo. After incubation with 10 μM A*β*(1–42) solution for 12 h at 25 °C, the functional surfaces covered with A*β* aggregates of distinct morphology were observed using AFM (Figure 8d–e). On the monovalent **G1** surface, A*β*(1–42) tended to form fibril-like aggregates, while on the trivalent **G3** surface, A*β*(1–42) tended to form globular aggregates. As expected, the aggregates with two morphologies coexisted on the divalent **G2** surface. These results are consistent with those proposed by Ban et al., who researched the morphology of A*β*(1–40) deposits on different sulfonated surfaces in 2006 [110]. Furthermore, Fourier transform reflection adsorption spectroscopy (FTIR-RAS) showed the altered secondary structure of A*β* modulated by multivalent sugars. In particular, the specific band assigned to the antiparallel *β*-sheet (approximately 1632 cm^−1^) was much weaker in the spectrum of **G3** surface than that of **G1** and **G2** surfaces, implying that the fibril-like aggregates were composed of more antiparallel *β*-sheets than globular deposits. The FTIR-RAS also showed a much larger intensity ratio of amide I to amide II in the spectrum of **G3** surface, which indicated the most specific orientation of A*β* to the substrate.

On the other hand, a previous study had revealed the possible binding region (HHQK) of A*β*(1–42) by using sulfonated sugars [111] and surmised that cationic His13, His14, and Lys16 could strongly interact with anionic sulfo through electrostatic interaction. In this work, the evaluation of SPR results demonstrated two types (1:1 and 2:1 binding modes) of the interaction between glycoclusters and A*β* peptides. For the **G1** surface, two independent sugars were bound to an A*β* peptide in the 2:1 mode, indicating a longer interaction distance between sugars with an interval of at least 2.2 nm. From the perspective of spatial matching, His13 and Lys16 (interval: 1.0–2.0 nm) were suggested as the most likely amino acids participating in this binding mode. By comparison, the sugar intervals of multivalent **G2** and **G3** were 0.5–1.0 nm, and matched with that of His13–His14 (interval: 0.8–1.0 nm), which induced a 1:1 binding mode between sugars and the His–His region because of the steric effect. Therefore, in this study, the altered binding mode mostly induced by steric hindrance probably resulted in the alternative morphology or secondary structure of A*β* aggregates, because the peptide has been reported to undergo conformation transition in response to ligand binding [112,113].

The nucleation phase is known as the rate-limiting step of the growth process of amyloid aggregates, and in vitro, the seeding of protein bulk solution with preformed fibrils is regarded as an effective strategy to dramatically accelerate fibril growth because of the accelerated nucleation phase [114]. Moreover, the seeds do not need to be composed of the same protein as the bulk solution [115]. Hence, the seed–water interface may account for this cross-seeding effect. However, comprehensive understanding of the molecular mechanism of fibril seeding is still a challenge, because the subject under investigation (i.e., protein) is highly complicated both in composition and structure. Therefore, simplified interfaces such as or peptide- or amino acid-modified surfaces may be the ideal platform for further investigation. As described in Section 4, Sun et al. [41,100] designed diverse chiral amino acid-modified surfaces and proposed the critical role of surface stereoselective interaction in A*β* aggregation. Lequin and Ongeri [116] designed a novel class of peptidomimetics composed of two hydrophobic dipeptides (Ala-Val and Val-Leu) linked to a d-glucopyranosyl scaffold. By combining targeted recognition interfaces (dipeptide–water) and hydrophilic sugar *β*-breakage strategy, these compounds considerably inhibited amyloid fibril formation, even at a low peptidomimetics to A*β* ratio of 0.1:1. Compellingly, this inhibition was found to be sequence-specific, because the interactions of these peptidomimetics with A*β* depended on their hydrophobic amino acid residues, which was proven by nuclear magnetic resonance (NMR) saturation transfer difference experiments. At these biomolecule–water interfaces, diverse driving forces are integrated together to modulate the behavior of protein aggregation, particularly these weaker interactions (i.e., hydrogen bonds and stereoselective or sterical effect) have become non-negligible.

## 6. Conclusions and Outlook

The demand for comprehensive understanding of the interfacial behavior of protein aggregation (e.g., amyloidosis), which distinctly differs from that in bulk solutions, has spawned a variety of frontier researches and discoveries, because of the extremely high reference value in pathological and therapeutic investigation of numerous critical diseases, particularly neurodegenerative diseases. In this review, we briefly introduced the objectives and implications of relevant research on the aggregation of proteins/peptides at artificial biointerfaces, analyzed diverse aggregation behaviors with the impact of given interfacial properties provided by artificial biomaterials (i.e., hydrophobic, charged, or chiral surfaces), revealing diverse driving forces (i.e., hydrophobic or electrostatic interaction, covalent bonds, hydrogen bonds, and stereoselective or sterical effect) that can strongly affect the different phases of protein aggregation, and summarized the recent progress in biomolecular surface-induced amyloidosis, emphasizing the critical role of weaker driving forces in fibril formation. Previous studies have revealed increasing prospects in this field, but numerous challenges remain unsolved.

The first challenge is how to dynamically monitor the aggregation process for the obtainment of real-time detailed information (i.e., morphography, components, or structures) on diverse aggregates in different phases. In the past decades, the development in this field was mostly based on the improvement of methodologies for aggregation studies, such as the technologies of AFM, SAXS, CD, SPR and TIRFM. For instance, previous studies about the impact of surface chemistry on the morphography of A*β* aggregates were mainly ascribed to the continuing development of in situ AFM technology [117,118]. Hence, the obtainment of comprehensive information on the aggregates is relied on the improvement and popularization of various in situ technologies (i.e., IR/Raman spectrum or SEM/TEM), enabling these technologies to be operated in solution and track the formation and fate of individual aggregates on surfaces, which may assist in obtaining more direct and abundant data about the components or spatial structures of these aggregates [119,120,121]. The second challenge is the research about weaker interactions (hydrogen bonds, stereoselective or sterical effect) between cytomembrane and proteins, which are hardly observed using common equipment and often neglected. However, several recent reports have indicated their critical roles in protein aggregation, attracting increasing interest from researchers in this field [96,98]. We anticipate that “smart polymers”, known as an excellent tool to realize the transformation from tiny changes into microscopic properties of modified surfaces [122,123,124], may possess its unique opportunities in this field and help further understand the impact of weaker interactions on protein aggregation. The third challenge is how to directly observe and elucidate the interfacial effect on protein aggregation in cells, where fibril formation is also strongly modulated by the complicated physicochemical environment (i.e., pH, ionic strength and diverse enzymes) provided by cytochylema. Furthermore, these physiological processes occurring in cells are dynamic and can hardly be traced using common technologies. By comparison, more in vitro experiments have been successfully performed to investigate the protein aggregation at artificial biointerfaces, as aforementioned in the text. However, the studies of pathogenic protein aggregation are initially aimed at the development of in vitro protein misfolding aggregation assays and subsequently offering comprehensive information for the early diagnostics and therapeutics in vivo. Therefore, the final and most pivotal challenge for present stages may be the guidance of experiments in vivo by using the results obtained in vitro. As the most typical biointerface in vivo, cell membrane is highly complex in terms of components and structures, thus the environment it can provide may extensively exceed our imagination. For example, the surface micro–nanostructure and corresponding steric hindrance [125,126], the membrane compounds (i.e., carbohydrates, lipids, proteins, etc. [127]), and their detailed mechanism of interactions with proteins have not been revealed clearly. On the other hand, cells can sense the physical or chemical properties of surrounding environment (e.g., substratum rigidity, roughness, and topography of recognition sites), and the membrane surfaces display continuous deformations of amplitude at the nanometer-level. Recent research has indicated that these surface undulations may result in the transient contact of membrane compounds with surroundings, generating forces of piconewton intensity as a result of rapid formation and dissociation of intermolecular bonds [128]. The effect of these discontinuous weak forces on protein aggregation has rarely been observed and demonstrated in in vitro experiments. Therefore, for improved mimicking of cell surfaces, more precisely controlled and compatible or dynamically tunable artificial biointerfaces are needed, which depends on the subtle design and the improvement of surface modifications [129].
